# Inborn Errors of Immunity in Pediatric Hematology and Oncology: Diagnostic Principles for Clinical Practice

**DOI:** 10.3390/jcm14176295

**Published:** 2025-09-05

**Authors:** Giulia Roberti, Giulia Maestrini, Beatrice Polito, Leonardo Amato, Eva Parolo, Gabriella Casazza, Rita Consolini, Giorgio Costagliola

**Affiliations:** 1 Section of Pediatric Hematology and Oncology, Azienda Ospedaliero-Universitaria Pisana, 56126 Pisa, Italy; roberti.giulia@hotmail.it (G.R.); giulia.mae13@gmail.com (G.M.); bea.polito95@gmail.com (B.P.); leonardoamato97@gmail.com (L.A.); eparolo89@gmail.com (E.P.); g.casazza@ao-pisa.toscana.it (G.C.); 2 Section of Clinical and Laboratory Immunology, University of Pisa, 56126 Pisa, Italy; rita.consolini@unipi.it

**Keywords:** autoimmune cytopenia, autoimmune hemolytic anemia, autoimmune lymphoproliferative immunodeficiencies (ALPID), autoimmune neutropenia, immune thrombocytopenia, lymphoma, polyclonal lymphoproliferation, malignancy, myelodysplastic syndromes, eosinophilia

## Abstract

Immune dysregulation is being increasingly recognized as a leading sign of a wide spectrum of inborn errors of immunity (IEIs). Therefore, patients with IEIs are frequently managed in non-immunological settings, including hematology and oncology units, during the diagnostic process or follow-up. The most relevant hematological signs associated with IEIs comprise autoimmune cytopenia (AIC), lymphoproliferative diseases (LPD), malignancies, hemophagocytic lymphohystiocitosis (HLH), bone marrow failure (BMF), myelodysplastic syndromes (MDS), and peripheral or tissue eosinophilia. The prognosis of patients with IEIs can significantly improve when a molecular diagnosis is established, as it can allow the use of targeted treatments, guide appropriate follow-up strategies and, in some cases, support the rationale for hematopoietic stem cell transplantation or gene therapy. Therefore, there is an urgent need to recognize the warning signs suggestive for an underlying IEI among patients presenting with common hematological features and to ensure an appropriate diagnostic approach. As a general rule, clinicians should always provide a clinical alert in the presence of two or more IEI-associated hematological signs, as well as a positive familial history for IEI or hematologic immune dysregulation, a personal history of severe infections, and other signs of immune dysregulation. Concerning AIC, an increased likelihood of IEI is characteristic of patients with treatment refractoriness, autoimmune hemolytic anemia, or multilineage cytopenia. In the case of LPD, the main elements of suspicion are represented by the chronic or recurrent disease course, the persistence of Epstein–Barr Virus (EBV) infection, and the development of lymphoproliferation in atypical localizations. Among patients with malignancy, clinicians should investigate for IEI those with rare neoplasia, virus-associated tumors, and an association with syndromic features, while patients with HLH should always receive an immunological assessment when a clear rheumatologic trigger, underlying malignancy, or well-recognized cause is not evident. The case of MDS and BMF is complex, as new monogenic entities are continuously being described. However, it is pivotal to consider the presence of monocytopenia, warts, vasculitis, and neurological disease, as well as specific cytogenetic abnormalities, such as chromosome 7 monosomy, as warning sings for IEIs. Finally, the main red flags for IEIs in patients with eosinophilia are skeletal/facial abnormalities, recurrent abscesses, refractory eczema, organomegaly, or thrombocytopenia.

## 1. Introduction

The phenotypic spectrum of inborn errors of immunity (IEIs) is in rapid expansion, and new monogenic entities are discovered every year. Non-infectious manifestations of IEI, which include autoimmunity, lymphoproliferation, atopy, malignancy, and others, are being increasingly recognized as presenting signs of a wide number of IEIs [1,2]. Therefore, an enhanced awareness about the warning signs to suspect an underlying IEI is warranted among different categories of physicians. The most common IEI-associated hematological features encompass autoimmune cytopenia (AIC), lymphoproliferative diseases (LPD), lymphoid or extra-lymphoid malignancies, hemophagocytic lymphohystiocitosis (HLH), bone marrow failure (BMF)/myelodysplastic syndromes (MDS), and peripheral or tissue eosinophilia [3]. As these clinical signs are extremely common in clinical practice, the primary challenge for physicians is the identification, among a wide number of patients, of those carrying a higher likelihood of an IEI and deserving dedicated investigations. Nowadays, the importance of reaching a molecular diagnosis in patients with IEIs that can present with hematologic immune dysregulation (H-ID) becomes more evident thanks to the availability of targeted therapies that open a new therapeutic scenario [4,5,6].

In this paper, we aim to discuss the most relevant IEI-associated hematological phenotypes and molecular pathways to identify some key parameters that should promptly lead to immunological assessment. We also discuss the clinical implications of the most relevant IEIs that can be encountered in the pediatric hematology and oncology setting and provide some key elements for the implementation of an appropriate diagnostic approach based on the hematological presenting signs.

For this purpose, we conducted a literature review on MEDLINE database in which the search terminology was constructed using the name of the specific IEI-associated hematological sign (AIC, LPD, BMF, MDS, eosinophilia, HLH, malignancy) and the terms “inborn errors of immunity” and “immunodeficiency.” The review included clinical trials, prospective studies, case series, and reports published in English up to July 2025.

## 2. Autoimmune Cytopenia and IEIs

Autoimmune cytopenias (AICs) are among the most common autoimmune manifestations of IEIs, especially in the pediatric population [7]. AICs include immune thrombocytopenic purpura (ITP), autoimmune hemolytic anemia (AIHA), autoimmune neutropenia (AIN), and Evans syndrome (ES)—a condition involving two or more simultaneous or sequential autoimmune cytopenias. AICs can represent the initial clinical manifestation of an underlying IEI, especially when cytopenias are multilineage, relapsing, or treatment-refractory. Specifically, from literature data, it emerges that AIC is 120-fold more common in patients with IEI when compared to the general population [8]. The frequency of AHA is 600-fold higher, therefore highlighting this presentation as a potential warning sign for an underlying IEI [8]. Moreover, recent studies also pointed out that around 50% of patients with ES present an underlying, genetically confirmed IEI [9]. Concerning ITP, early chronic disease and treatment refractoriness have been associated with an increased likelihood of IEI [10], while AIN, although commonly idiopathic and self-limiting, can be associated with germline IEI variants, especially in patients with late-onset (onset at age > 5 years) or long-lasting (duration > 36 months) neutropenia [11].

The most common IEIs associated with AICs are those included in the autoimmune lymphoproliferative immunodeficiencies (ALPID) spectrum, other immune dysregulation disorders, primary antibody deficiencies (common variable immunodeficiency [CVID] and related disorders), and syndromic IEIs (such as the 22q11.2 deletion syndrome, Table 1) [10,12,13]. These conditions present with a complex clinical phenotype; therefore, following the onset of AICs, additional manifestations may occur, such as LPD, other autoimmune features, atopy, and more, as discussed further below. For this reason, providing a diagnosis is of primary importance to initiate an appropriate follow-up and, when needed, specific medical treatment.

The pathogenesis of AICs in the context of IEIs is complex and involves multiple mechanisms of immune dysregulation, which depend on the underlying germline defect and associated molecular pathways. In some monogenic diseases, the loss of self-tolerance depends on genetic defects affecting key immunoregulatory pathways, including those implicated in regulatory T cells (Treg) function (CTLA4 and LRBA haploinsufficiency, STAT3 gain-of-function [GOF]), lymphocyte proliferation (PI3K), or lymphocyte apoptosis (FAS and related genes) [14,15]. These alterations can severely compromise immune checkpoints, regulatory T cell function, and the balance between effector and suppressor lymphocytes, thus finally promoting autoreactivity. Autoantibody production with subsequent destruction of blood cells is not the only effector mechanism responsible for AIC. Indeed, T cell-mediated cytotoxicity, complement activation, splenic sequestration, myelosuppression, and bone marrow failure can contribute to the pathogenesis of cytopenia. In the specific case of ALPS, the defective FAS-mediated apoptosis leads to the accumulation of self-reactive lymphocytes and autoimmune destruction, resulting in a complex picture of AIC and LPD [14,15].

There is increasing interest in searching for predictors of an underlying IEI in patients presenting with AIC. As a general rule, AICs associated with IEIs tend to present with a chronic or relapsing course and poor response to conventional treatments such as steroids or intravenous immunoglobulin (IVIG) [12]. It is also important to note that in several cases, these patients may not have a prior history of infections at diagnosis, delaying the suspicion of immunodeficiency [13,16].

It is worth emphasizing that immunophenotyping often reveals characteristic abnormalities in patients with AIC secondary to IEIs. These features have been described in recent studies and include increased double-negative T cells (DNTs), decreased switched memory B cells, or altered Treg number or function; therefore, their identification can help clinicians refine their diagnostic approach [15].

## 3. Polyclonal Lymphoproliferation and IEIs

Polyclonal (benign) lymphoproliferation (pLPD) may manifest as chronic or recurrent lymphadenopathy, hepatomegaly, splenomegaly, infiltration of non-lymphoid tissues, and/or elevated lymphocyte counts in peripheral blood [17]. These clinical and laboratory findings can represent part of the phenotypic spectrum of IEIs, including those associated with immune dysregulation and autoinflammatory disorders (Table 2). They may occur as initial presentations or arise during the chronic course of disease [2].

Among IEIs potentially presenting with lymphoproliferation, the most relevant are those of the ALPID spectrum, common variable immunodeficiency, APDS, EBV-related disorders, and others [18].

CVID is featured by an extremely heterogeneous clinical phenotype, potentially presenting with different features of recurrent/severe infections, autoimmunity, granulomatous disease, and pLPD [19]. In this condition, laboratory assessment typically shows reduced serum IgG and IgA concentration (with or without low IgM), impaired vaccine responses, or decreased switched memory B cells, in the absence of profound T-cell deficiency [20]. In CVID, pLPD may appear as benign chronic lymphadenopathy and hepatosplenomegaly, but is also associated with increased malignancy risk, particularly lymphomas [21]. Different studies estimated a prevalence of lymphoma of 8–15% in CVID patients [22,23], and this represents the second leading cause of mortality after chronic lung disease. The pathogenesis of pLPD in CVID patients is not fully understood, and specific immunological markers are lacking. However, data from large cohort studies (such as the EUROclass study) suggest associations with expanded transitional B cells and CD21^low^ B cells [8]. Mutations in *NFKB1*, *TACI*, and other genes have also been linked to autoimmunity and pLPD in CVID [24,25]. Histologically, benign lymphadenopathy in CVID may mimic lymphoma [26], due to disrupted germinal centers and clonal B-cell expansions [27]. This diagnostic ambiguity often necessitates an excisional biopsy. Moreover, CVID patients with lymphoproliferation or granulomatous disease are at increased risk for granulomatous–lymphocytic interstitial lung disease (GLILD), a serious pulmonary manifestation associated with higher morbidity [28].

Beyond CVID, pLPD is often the first disease sign or leading phenotypic feature of ALPS and related disorders. This condition is characterized by chronic lymphoproliferation and hematologic autoimmunity, caused by mutations in the FAS pathway (FAS, FASL, CASP10), resulting in the accumulation of αβ DNTs. Most patients develop persistent lymphadenopathy, splenomegaly, and autoimmune cytopenias and also have an increased risk of lymphoma. Diagnosis of ALPS relies on clinical features, elevated DNTs, elevated soluble FASL, vitamin B12, interleukin 10 (IL-10), IL-18, and impaired FAS-mediated apoptosis [29]. Other diseases of the ALPID spectrum, including CTLA4 and LRBA haploinsufficiency, STAT3 GOF, and NFKB1 mutations, can present with pLPD as a prominent disease feature [30,31].

**Table 2:** Key Features Suggestive of IEIs in Patients with Non-Infectious Lymphoproliferation [17,18,30,31].

Associated Feature	Indicative of/Frequently Found in	Notes
Autoimmune cytopenias	ALPS, CVID, DADA2, CTLA-4/LRBA haploinsufficiency, STAT3 GOF	Frequent early clue; particularly if multilineage
Enteropathy	IPEX, CVID, CTLA-4/LRBA d haploinsufficiency, STAT3 GOF	Suggests immune dysregulation
Endocrinopathy (e.g., type 1 diabetes, thyroiditis)	IPEX, CTLA-4/LRBA haploinsufficiency	Especially if in association with cytopenias or lymphadenopathy
Recurrent/severe infections	CVID, APDS, XMEN, CTLA-4 haploinsufficiency	Suggests impaired immune protection
Eczema or eczematous dermatitis	STAT3 GOF, IPEX, WAS,	Particularly if early-onset and severe
Granulomatous disease (lung, spleen, liver)	CVID (GLILD), CTLA-4 haploinsufficiency	Often coexists with lymphadenopathy or splenomegaly
Neurological involvement	CTLA-4 haploinsufficiency, STAT1 GOF, STAT3 GOF, APDS	Uncommon in infection-driven lymphoproliferation
Family history of immunodeficiency/autoimmunity	ALPS, STAT3 GOF, CVID, CTLA-4/LRBA haploinsufficiency, APDS	Strong clue; especially in early-onset presentations
Marked hypergammaglobulinemia or hypogammaglobulinemia	CVID, ALPS, APDS, DADA2, CTLA-4 haploinsufficiency	Quantitative immunoglobulin abnormalities support IEI suspicion
Elevated αβ double-negative T cells (DNTs)	ALPS (and other diseases of the ALPID spectrum), RALD	Key diagnostic biomarker

Abbreviations: ALPS = autoimmune lymphoproliferative syndrome; ALPID = autoimmune lymphoproliferative immunodeficiencies; APDS = activated PI3K-delta syndrome; CTLA-4 = cytotoxic T-lymphocyte antigen 4; CVID = common variable immunodeficiency; DADA2 = deficiency of adenosine deaminase 2; IPEX: Immune Dysregulation, Polyendocrinopathy, Enteropathy, X-Linked Syndrome; LRBA = lipopolysaccharide-responsive beige-like anchor protein; RALD = RAS-associated autoimmune leukoproliferative disease; STAT = signal transducer and activator of transcription; WAS: Wiskott-Aldrich syndrome; XMEN: X-linked immunodeficiency with magnesium defect, Epstein–Barr virus (EBV) infection, and neoplasia.

Activated PI3Kδ Syndrome (APDS) is a condition situated at a clinical crossroad between ALPIDs and CVID-like disorders. In this disease, GOF mutations in the PIK3CD gene or loss-of-function mutations in the PIK3R1 gene lead to hyperactivation of the PI3K–mTOR pathway [32]. Over 50% of patients show chronic lymphoproliferation, and hematologic malignancies occur in 12–13%, with some cohorts reporting up to 28% [33,34].

EBV-associated lymphoproliferation is observed in immunodeficiencies such as X-linked proliferative syndromes (XLP), ITK, STK4, and RASGRP1 deficiencies, and X-linked immunodeficiency with magnesium defect, Epstein–Barr virus (EBV) infection, and neoplasia (XMEN), and is marked by chronic lymphadenopathy, persistent viremia, and increased risk of EBV-driven lymphomas and HLH (see specific section) [35].

Rare associations with chronic or recurrent lymphoproliferation include Wiskott–Aldrich syndrome, which may show lymphoid malignancies but rarely pLPD [36]. Phenocopies of IEIs, such as NRAS or KRAS mutations causing RAS-associated autoimmune leukoproliferative disease (RALD), can mimic classical ALPS presenting with lymphadenopathy, cytopenias, and increased risk of lymphoma. [18,37]. Concerning other immune dysregulation disorders, lymphadenopathy occurs in 15% of cases of Immune Dysregulation, Polyendocrinopathy, Enteropathy, X-Linked Syndrome (IPEX) syndrome (caused by FOXP3 mutations), with lymphoma being rare [18]. Finally, in patients with deficiency of adenosine deaminase 2 (DADA2, caused by CECR1 mutations), splenomegaly is seen in 30% and lymphadenopathy in about 10% of cases, and patients can exhibit ALPS-like phenotypes. Notably, this condition can also present overlapping features with BMFs, as further discussed [38,39].

## 4. Malignancy and IEIs

Individuals with IEIs have an increased risk of developing both hematological and solid malignancies. Malignancies represent the second leading cause of death in patients with IEI after infections [40,41], and the overall cancer risk in children with IEI is estimated to range between 4% and 25% [42]. Several mechanisms are linked to cancer development in this individual with IEI. Among them, intrinsic factors include impaired cell-mediated immune surveillance, dysfunctional differentiation and activity of dendritic cells, and defects in DNA repair pathways, while extrinsic factors involve oncogenic infections (HPV and EBV) and chronic tissue inflammation [43,44].

The most common malignancies in children with IEI are represented by lymphomas. Mature B-cell non-Hodgkin lymphoma (NHL) is the most frequent diagnosis, with diffuse large B-cell lymphoma occurring more often than Burkitt lymphoma [45]. The IEI subtype and the patient’s age significantly influence the type of malignancy that may develop, depending on the underlying molecular defect; thus, the specific IEI subtype could help guide early screening, tailored treatment, and surveillance strategies (Table 3) [44].

Regarding the overall frequency of malignancies in the specific IEIs, it is noteworthy that DNA repair disorders—such as ataxia–telangiectasia (AT), Bloom syndrome, and especially Nijmegen breakage syndrome—carry the highest risk of cancer [44,45,46,47]. In these conditions, lymphomas and lymphoblastic leukemia tend to occur more frequently and at a younger age than in the general population [40,46]. However, in clinical practice, due to the relative frequency of the different IEIs, most IEI-associated malignancies are observed in patients diagnosed with CVID or immune dysregulation disorders. These conditions are mostly associated with an increased incidence of lymphoma, especially those linked to EBV. In addition, patients with phagocyte disorders and bone marrow failure syndromes are at increased risk of developing MDS and acute myeloid leukemia (AML) [48].

**Table 3:** Most common associations between IEIs and specific malignancies [18,44,45,47].

IEI Category	Most Common Diseases	Overall Risk of Malignancy	Association with Malignancies
DNA repair disorders	Ataxia–telangiectasia	16%	Lymphomas (>NHL, MALT lymphomas, extranodal marginal zone lymphomas); breast cancer, liver cancer, gastrointestinal malignancies
	Nijmegen breakage syndrome	50%	Lymphomas (>NHL), ALL
			AML
	Bloom syndrome	25%	Leukemia, lymphoma, skin cancers
Primary antibody deficiencies	CVID	9%	NHL, HL, gastric cancer, breast cancer
	XLA	2–6%	NHL, gastrointestinal malignancies
Immune dysregulation disorders and combined immunodeficiencies	Disorders of the ALPID spectrum	Variable, depending on the genetic background (5–17%)	Lymphomas (>HL)
	APDS	13–28%	Lymphomas (>NHL)
	XLP-1 and other EBV-associated disorders	30% (for XLP)	EBV-associated lymphoma
Inherited bone marrow failure syndromes and phagocyte disorders	Severe congenital neutropenia	6–7%	MDS, AML
	Fanconi Anemia	27%	MDS, AML, ALL, head and neck malignancies, liver malignancies
	GATA2 deficiency	>50%	MDS, AML
IEI with syndromes	WAS	12%	Lymphomas (>NHL), leukemia

Abbreviations: ALPID = autoimmune lymphoproliferative immunodeficiencies; ALL: acute lymphoblastic leukemia; AML: acute myeloid leukemia; APDS = activated PI3K-delta syndrome; CVID = common variable immunodeficiency; HL: Hodgkin lymphoma; MDS: myelodysplastic syndrome; NHL: Non-Hodgkin lymphoma; XLA: X-linked agammaglobulinemia; XLP-1: X-linked proliferative syndrome 1; WAS: Wiskott–Aldrich syndrome.

Diagnosing malignancy in patients with IEI can be particularly challenging due to underlying comorbidities and overlapping inflammatory, infectious, or lymphoproliferative processes. In some cases, the IEI diagnosis precedes the onset of cancer, but in other children, malignancy may be the initial manifestation of the underlying immunodeficiency, leading to misdiagnosis or diagnostic delays [47]. When malignancy is the first disease presentation, several warning signs can lead to the diagnosis of an IEI. These include tumor-associated features (younger age compared with sporadic cases, rarity, atypical areas or histology, virus-associated neoplasia) and clinical phenotype (i.e., association with neurological disease, previous history of polyclonal LPD, features of infections or immune dysregulation, records of consanguinity or early childhood death in the family) [47].

The diagnosis of an underlying IEI in a child with malignancy carries significant clinical implications regarding treatment and follow-up strategies. Indeed, IEIs can significantly affect the safety and efficacy of conventional cancer therapies, and overall survival following cancer is lower in patients with IEIs compared to the general population. An example is represented by lymphomas, as survival after lymphoma in IEI patients is reported at 62%, mainly due to comorbidities (such as infections) and severe treatment-related toxicity [40].

Moreover, IEIs associated with DNA repair defects (and especially AT) cause a higher sensitivity to ionizing radiation; this can influence treatment strategies (including the avoidance or dose-reduction, when feasible, of radiotherapy-based regimens), and follow-up protocols, in which the use of PET/CT could require limitations [47,49]. In selected IEIs, early hematopoietic stem cell transplantation (HSCT) should be considered, as it may restore immune surveillance and consequently reduce cancer risk. HSCT may also serve to control malignancy and treat the underlying IEI [49,50].

## 5. Bone Marrow Failure, Myelodysplastic Syndromes, and IEIs

**Figure 1:**
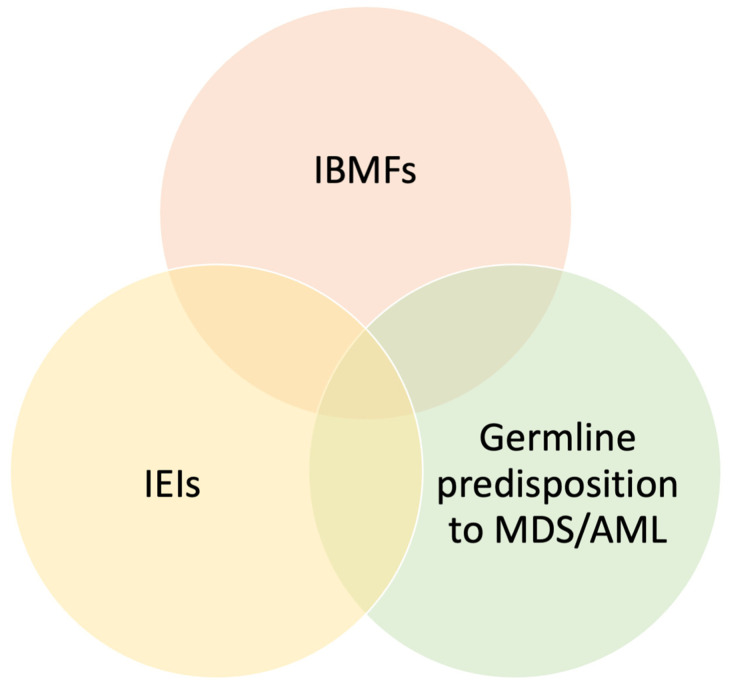
Overlaps between inherited bone marrow failure syndromes, inborn errors of immunity, and diseases with germline predisposition to myelodysplastic syndromes and acute myeloid leukemia. Abbreviations: IEIs = inborn errors of immunity; IBMFs = inherited bone marrow failure syndromes; MDS = myelodysplastic syndromes; AML = acute myeloid leukemia.

In the last few years, the molecular, pathogenic, and clinical relationship between IEIs, inherited bone marrow failure syndromes (IBMFs), and germline predisposition to MDS has been increasingly investigated (Figure 1). Current research is pointing out an immune impairment with a tendency towards immune dysregulation in the most studied IBMFs and acquired BMFs [51], such as Fanconi anemia, Diamond–Blackfan anemia, Shwachman–Diamond syndrome, and dyskeratosis congenita. This assumption supports the hypothesis of the existence of common molecular pathways and partly overlapping clinical phenotypes, thus also allowing the inclusion of IBMFs in the current IEI classification [52].

Some paradigmatic conditions with a clinical spectrum ranging across these entities have been recently characterized and include GATA2 deficiency, SAMD9-SAMD9L deficiency, DADA2, and others. A common element of these diseases is the coexistence of an immune defect in the context of a hypocellular bone marrow or a germline predisposition to MDS or myeloid malignancies. Since their clinical presentation can be non-specific at disease onset (i.e., with isolated cytopenia), it is essential to identify the red flags that may lead to the diagnosis of these potentially life-threatening disorders [53,54,55].

GATA2 deficiency is one of the most studied diseases. Its clinical spectrum includes neutropenia with low monocytes, reduced B-cell and NK-cell count, increased susceptibility to specific infections (HPV infections and HPV-related neoplasia, warts, mycobacterial infections), immune dysregulation, lymphedema, pulmonary disease (including pulmonary alveolar proteinosis), and increased risk of MDS [56,57]. Immune dysregulation is expressed through an increased risk of HLH-like febrile episodes, vasculitis, and connective tissue disease [58,59]. Notably, patients with GATA2 deficiency and MDS commonly have chromosome 7 monosomy in bone marrow cells (reported in up to 80% of cases) [56].

SAMD9 and SAMD9L deficiency show a clinical phenotype that partly overlaps with GATA2 deficiency [60]. Indeed, bone marrow hypocellularity and predisposition to MDS are the most common signs of these conditions. On the other hand, the immunological impairment is extremely variable in terms of clinical/laboratory expression and severity and is less well characterized [53]. Some reports describe GATA2 deficiency-like immunological abnormalities, including hypogammaglobulinemia and reduced B and NK cells, as well as severe infections [53].

Some patients, and especially those with SAMD9 mutations, can present with multiple abnormalities, which are included in the spectrum of the MIRAGE syndrome (myelodysplasia, infection, restriction of growth, adrenal hypoplasia, genital phenotypes, and enteropathy) [61]. Concerning karyotype, it is noteworthy that up to 50% of children and adolescents with MDS and chromosome 7 monosomy display a germline variant in GATA2 or SAMD9/SAMD9L [53,62].

Another relevant condition situated at a crossroad between IEIs and BMFs is DADA2. This disease has a wide phenotypic spectrum, which depends on the residual ADA2 activity. Patients with an inflammatory phenotype can develop vasculitis (also with panarteritis nodosa-like presentation), aphthosis, autoimmunity, and lymphoproliferation, as well as early thrombosis [63]. However, in some cases, the first or leading sign of DADA2 is represented by pure red cell aplasia or a complex picture of bone marrow hypocellularity [38]. Specific assays to determine ADA2 activity are available, and delivering a prompt diagnosis could be clinically relevant since the use of etanercept has been demonstrated to be specifically effective in the treatment of DADA2 patients [64].

However, the genetic background underlying IBMFs and predisposition to MDS is largely unexplored. New entities are being continuously described, and their characterization will hopefully lead to the earlier identification of patients at risk of progression to MDS or AML.

## 6. HLH and IEIs

Hemophagocytic lymphohistiocytosis (HLH) is a rare, potentially life-threatening clinical entity characterized by a severe impairment of immune homeostasis with uncontrolled and excessive inflammatory activation and cytokine secretion. HLH can be caused by primary alteration of the cytolytic pathway (with consequent T-cell expansion and cytokine production) or macrophage hyperresponsiveness, which can be triggered by a wide number of extrinsic factors [65].

Clinically, the most relevant signs of HLH include fever, splenomegaly, and cytopenia. Laboratory testing commonly shows elevated serum ferritin (often in association with low erythrocyte sedimentation rate) and triglycerides, low fibrinogen, and increased IL-2 receptor, while the demonstration of bone marrow hemophagocytosis is available only in a subset of patients [66].

Due to its diverse pathogenic pathways, HLH may represent a potential clinical manifestation of several diseases, including infections, malignancies, rheumatologic disease, and IEIs [66,67].

HLH occurring in patients with known mutations in genes related to granule-dependent cytotoxicity is termed “primary” or familial HLH (FHL). The current International Union of Immunological Societies (IUIS) classification distinguishes two subgroups of FHL based on the presence of hypopigmentation [52]. The first subgroup includes pathogenic variants in PRF1, STX1, UNC13D, STXBP2, FAAP24, SLC7A7, and RHOG, while the second subgroup consists of gene variants associated with Chediak-Higashi syndrome, Griscelli syndrome type 2, and, less commonly, Hermansky-Pudlak syndrome types 2 and 10 (Table 4) [52]. On the other hand, when HLH is triggered by infections, autoimmune diseases, or malignancy in the absence of specific mutations in FHL-related genes, it is termed “secondary” or “acquired”. However, this classification carries some limitations, since primary HLH is often triggered by infections and secondary HLH might develop on an unmasked pathologic genetic background [68,69].

Moreover, since both primary and secondary HLH stem from a loss of immune homeostasis, several IEIs other than FHL could predispose to HLH, with variable pathogenic mechanisms. A paradigmatic example is EBV-related disorders, where a defective control of the viral infection finally leads to EBV-associated lymphoproliferation and increased risk of virus-triggered HLH. Among these, one of the most studied diseases is XLP-1, in which HLH often represents the initial clinical manifestation [70] and in which the defective control of the viral infection finally leads to EBV-associated lymphoproliferation and increased risk for virus-triggered HLH [71,72].

A recent systematic review by Ricci et al. specifically focused on the analysis of IEI-associated HLH. The authors identified 178 patients with HLH linked to 46 distinct IEIs, which included CID, immune dysregulation disorders (such as XLP-1 and XLP-2), and phagocyte defects [73]. Notably, in 75% of cases, HLH preceded the IEI diagnosis, often with an unrecognized history of severe infections, thus underscoring the need for an increased awareness about the possibility of diagnosing IEIs in patients presenting with HLH [73].

Recognizing HLH as a potential presentation of an underlying IEI is pivotal for accurate diagnosis and effective treatment, especially during its acute onset. Treatment approaches vary depending on the underlying condition and the heterogeneity of disease-driving factors. While FHL often requires uniform and aggressive immunosuppressive therapy, as standardized in international treatment protocols, HLH associated with other IEIs may not [74]. In this context the term “HLH mimics” refers to a wide range of conditions that fulfill HLH criteria but do not benefit from standard HLH protocols, as they are caused by different molecular pathways [75]. Several IEIs can present as HLH mimics, including diseases with impaired control of the inflammatory response (autoinflammatory disorders, DADA2, and complex diseases such as GATA2 deficiency), conditions within the ALPID spectrum (i.e., APDS, STAT3 GOF), and others [53,63]. For these patients, targeted therapies directed against the underlying molecular defect are emerging as promising alternatives to conventional chemotherapy regimens [74], thus underscoring that the precise identification of HLH etiology is essential for improving outcomes.

## 7. Eosinophilia and IEIs

Peripheral eosinophilia is defined by an eosinophil count > 500/µL, and it is classified into mild (500–1500/µL), moderate (1500–5000/µL), or severe (>5000/µL); the term “hypereosinophilia” is used to identify conditions in which a blood eosinophil count >15,000/µL is reported in at least two different samples with a minimum interval of 4 weeks [76]. Eosinophilia is a common finding in clinical practice, and the most frequent causes are presented by allergy (even mild) and non-specific infections, although some parasitic infections can also trigger peripheral eosinophilia. However, in some cases, eosinophilia may be the presenting feature of broader conditions, including hematological malignancies, vasculitis, and IEIs.

Concerning IEIs (Table 5), eosinophilia is part of the clinical spectrum of most of the diseases classified into the spectrum of primary atopic disorders (PAD) [77]. These conditions, which are featured by immune dysregulation expressed mostly through atopy, include hyper-IgE syndromes (HIES), disorders of Tregs (such as IPEX and related disorders), Wiskott–Aldrich syndrome, Omenn syndrome, and others [78]. Given their presentation, PADs are often misdiagnosed, delaying the suspicion of IEI. It is not uncommon that patients with highly suggestive clinical phenotypes undergo hematological assessment for the persistence of peripheral eosinophilia. Moreover, since eczema itself can be associated with peripheral eosinophilia, identifying some specific IEI-associated findings may help guide the diagnostic process and avoid under-recognition of IEIs [78,79,80].

The case of HIES (most commonly caused by LOF mutations in STAT3 or DOCK8) is of paradigmatic relevance [81]. Patients with STAT3 HIES present with complex clinical phenotypes encompassing severe eczema, increased susceptibility to fungal infections (mucocutaneous candidiasis), skin abscesses, recurrent upper respiratory and sinopulmonary infections, skeletal (scoliosis, pathologic fractures) abnormalities, characteristic facies with high palate, and tooth retention. In this condition, laboratory findings typically point out peripheral eosinophilia and increased serum IgE concentration [82]. In patients with DOCK8 HIES, the association with neurological diseases is also commonly observed, as well as an increased risk for malignancy [78]. Specific scores to improve the recognition of HIES and also to suspect the underlying molecular defect are available [83].

Similarly, the IPEX spectrum has some relevant clinical peculiarities. These include the presence of eczema, enteropathy, endocrine autoimmune diseases, and increased susceptibility to infections. In this condition, caused by FOXP3 mutations, the main pathogenic driver is the reduced number/function of Tregs [84,85]. Other diseases with impairment of Treg function and peripheral immune tolerance can present with IPEX-like phenotypes, including peripheral eosinophilia. These comprehend STAT3 GOF mutations, STAT1 GOF mutation, STAT5b deficiency, CD25 deficiency, CTLA4 haploinsufficiency, and LRBA haploinsufficiency [84,86]. Notably, in these conditions, the clinical expression is extremely variable, as patients can develop features partly overlapping ALPID (AIC, LPD), IPEX, and CVID phenotypes [87].

In patients with WAS, the classic clinical triad includes eczema, thrombocytopenia, and immunodeficiency. In this case, the association between eosinophilia and thrombocytopenia with small-sized platelets can significantly help in orienting the diagnostic process [88].

Ultimately, patients with OS develop an early severe phenotype of SCID associated with hepatomegaly, splenomegaly, diffuse lymphadenopathy, and eczema, in which a Th2 expansion is observed, thus leading to peripheral eosinophilia [89]. In these patients, differential diagnosis should also consider skin barrier defects, such as Comel–Netherton syndrome and severe dermatitis, multiple allergies, and metabolic wasting syndrome (SAM), and others [79].

**Table 5:** Most frequent inborn errors of immunity associated with peripheral eosinophilia [77,78,79,81,84].

Clinical Phenotype	Specific Disease	Most Relevant Associated Signs
**HIES**	STAT3-HIES	Severe eczema Fungal infections Skin abscesses Recurrent pneumonia Scoliosis Tooth retention High palate
	DOCK8-HIES	Severe eczema Skin abscesses and recurrent cutaneous infections Recurrent sinopulmonary infections Fungal infections Allergies Risk of malignancy Risk of neurological complications (i.e., cerebral vasculitis)
**IPEX and related phenotypes**	IPEX	Endocrinopathy Enteropathy Eczema Autoimmune cytopenia
	STAT3 GOF	Autoimmune cytopenia Lymphoproliferation Multiorgan autoimmunity Enteropathy
	STAT1 GOF	IPEX-like phenotype Autoimmunity (endocrinopathy, arthritis) Predisposition to mucocutaneous candidiasis
	CTLA4 haploinsufficiency	Autoimmune cytopenia Lymphoproliferation GLILD Enteropathy Respiratory infection Other autoimmune diseases (thyroiditis, arthritis, uveitis)
	LRBA haploinsufficiency	Autoimmune cytopenia Lymphoproliferation Enteropathy Autoimmunity (hepatitis, uveitis, diabetes) Respiratory infections, bronchiectasis GLILD
**Syndromic IEIs**	Wiskott–Aldrich syndrome	Thrombocytopenia Eczema Lymphopenia
**Combined immunodeficiencies**	Omenn syndrome	Early onset of life-threatening infections Splenomegaly, hepatomegaly, Generalized erythroderma
**Cutaneous diseases**	Comel–Netherton syndrome	Severe eczema Congenital ichthyosis Bamboo hair

CTLA4 = cytotoxic T-lymphocyte antigen 4; DOCK8: Dedicator of Cytokinesis 8; HIES: hyper-IgE syndrome; IPEX: Immune Dysregulation, Polyendocrinopathy, Enteropathy, X-Linked Syndrome; LRBA = lipopolysaccharide-responsive beige-like anchor protein; STAT3/STAT1 = signal transducer and activator of transcription 3/1.

## 8. Practical Implications

**Figure 2:**
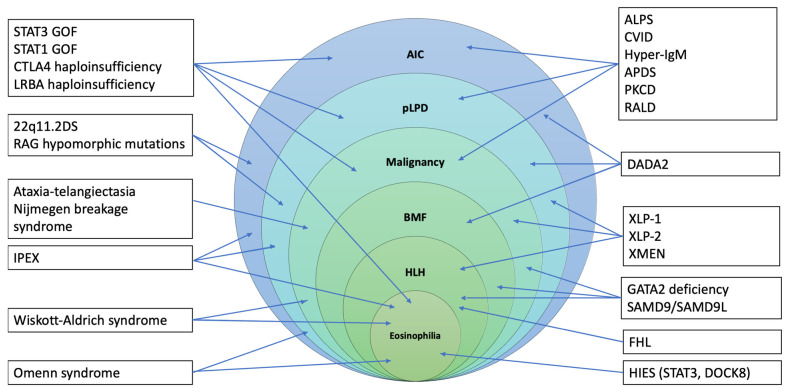
Clinical overlaps between IEIS presenting with hematological features. Abbreviations: 22q11.2DS = 22q11.2 deletion syndrome; ALPS = autoimmune lymphoproliferative syndrome; APDS = activated PI3K-delta syndrome; CTLA4 = cytotoxic T-lymphocyte antigen 4; CVID = common variable immunodeficiency; DADA2 = deficiency of adenosine deaminase 2; IPEX: Immune Dysregulation, Polyendocrinopathy, Enteropathy, X-Linked Syndrome; LRBA = lipopolysaccharide-responsive beige-like anchor protein; PKCD = protein kinase C delta; STAT3/STAT1 = signal transducer and activator of transcription 3/1; RALD = RAS-associated autoimmune leukoproliferative disease.

Literature on the prevalence and treatment of IEIs associated with hematological involvement is rapidly expanding. Moreover, studies directly investigating the relevance of some immunological parameters (conventional and extended lymphocyte phenotyping) in identifying or predicting the development of H-ID have shed light on some of the core mechanisms driving its pathogenesis [14,90]. Despite this, clinical practice remains highly heterogeneous in terms of the identification of patients at risk for IEI, first and second-level laboratory assessment, and clinical management. Furthermore, as the most common IEI presenting with hematologic features can show a significant phenotypic overlap [91] (Figure 2), the diagnostic process is complex, and the identification of a molecular diagnosis can require a long time.

Providing a molecular diagnosis of IEI is of pivotal importance in patients with hematological diseases, since specific conditions may be eligible for targeted therapies, which include abatacept for CTLA4 or LRBA haploinsufficiency, leniolisib for APDS, or JAK inhibitors for STAT3-GOF [13,14]. Moreover, identifying an underlying IEI can lead to prompt HSCT in certain patients, to prevent further complications and improve the overall outcome [92].

### 8.1. Diagnosing IEIs in Patients with Hematological Diseases

#### 8.1.1. Warning Signs for IEIs in Patients with Hematological Diseases

**Figure 3:**
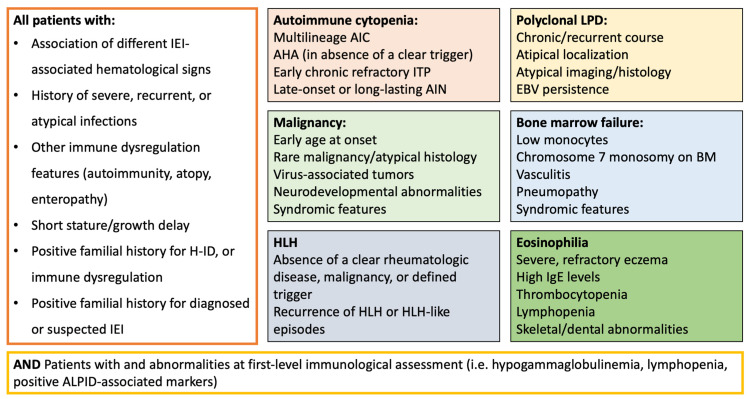
Warning signs for IEIs in patients with hematological diseases. Abbreviations: AIC = autoimmune cytopenia; AHA = autoimmune hemolytic anemia; AIN = autoimmune neutropenia; ALPID = autoimmune lymphoproliferative immunodeficiencies; BM = bone marrow; EBV = Epstein–Barr virus; HLH = hemophagocytic lymphohistiocytosis; IgE = immunoglobulin E; IEIs = inborn errors of immunity ITP = immune thrombocytopenic purpura; LPD = lymphoproliferative disease.

From the evidence discussed in this paper, it is possible to propose some clinical parameters that can promptly alert the clinician towards the possibility of an underlying IEI, even before performing dedicated laboratory investigations (Figure 3). Specifically, the association between two or more IEI-associated hematological signs (i.e., AIC and pLPD), the presence of other signs of immune dysregulation (atopy, autoimmunity, autoinflammation), as well as a positive familial history or a demonstrated increased susceptibility to infections, should be investigated in all patients presenting with hematological features.

Concerning patients presenting with AIC, clinicians should be alerted by multilineage cytopenia, refractory disease, early chronic ITP, or late-onset/long-lasting AIN, while AHA should always be considered suspect for IEI unless other causes (i.e., specific infections) have been identified. In patients with pLPD, IEI should be considered in case of chronic/recurrent course, involvement of atypical localization (i.e., GLILD), and EBV persistence, as well as a positive personal or familial history for malignancy, pLPD, or HLH [93]. Similarly, patients with malignancy should undergo immunological assessment in the presence of rare neoplasia (localization, histopathology), virus-associated tumors, association with neurological or neurodevelopmental abnormalities, syndromic features, and atypical treatment toxicity or refractoriness. On the other hand, screening for IEI is strongly suggested in all pediatric patients presenting with HLH when a clear rheumatologic disease or malignancy is excluded or unlikely [94]. Moreover, EBV-associated HLH is a relevant warning sign for several IEIs, including XLP syndromes [95].

Among the wide number of patients with MDS or BMF, the main IEI-associated signs are represented by low monocyte count, pneumopathy, vasculitis, and chronic infection (HPV-associated infection, chronic mycobacterial disease), as well as specific karyotype aberrations (chromosome 7 monosomy). Ultimately, in patients with eosinophilia, the main warning signs encompass the association with thrombocytopenia, high IgE concentration, lymphopenia, hypogammaglobulinemia, skeletal abnormalities, endocrinopathy, and enteropathy.

#### 8.1.2. An Overview of the Diagnostic Approach

Based on the evidence discussed above, we suggest conducting a baseline immunological assessment in all patients in whom clinical screening points out one of the IEI-associated warning signs. Notably, a negative first-line immunological evaluation does not rule out a potential diagnosis of IEI, particularly when clinical suspicion is high. Moreover, treatments for malignancies or immune dysregulation can compromise the reliability of most of the laboratory investigations (i.e., lymphocyte phenotyping and proliferation) and significantly influence the clinical expression of IEIs, thus underscoring the importance of a prompt identification of these red flags to guide early diagnostic assessment.

**Figure 4:**
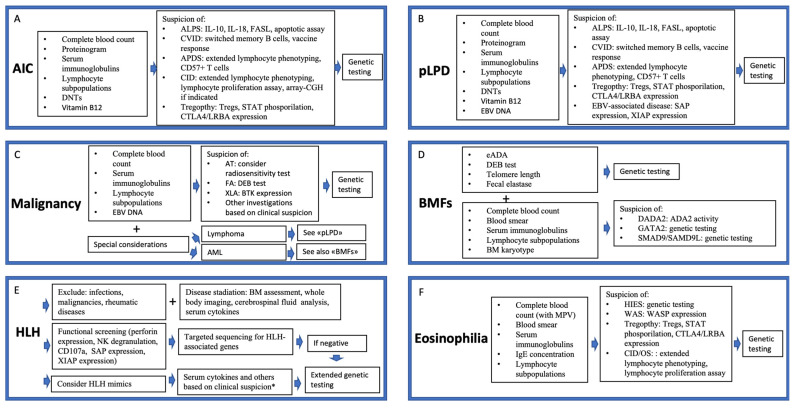
Proposed diagnostic approach for patients with hematological involvement and suspected inborn errors of immunity. (**A**): Approach for patients with AIC and suspected IEI. (**B**): Approach for patients with polyclonal lymphoproliferative disease and suspected IEI; (**C**): Approach for patients with malignancy and suspected IEI; (**D**): Approach for patients with bone marrow failure syndromes and suspected IEI; (**E**): Approach for patients with hemophagocytic lymphohistiocytosis and suspected IEI; (**F**): Approach for patients with eosinophilia and suspected IEI Abbreviations: ADA2: adenosine deaminase 2; AIC = autoimmune cytopenia; ALPS = autoimmune lymphoproliferative syndrome; AML: acute myeloid leukemia; APDS = activated PI3K-delta syndrome; BM = bone marrow; BMFs: Bone marrow failure syndromes; BTK: Bruton tyrosine kinase; CID: combined immunodeficiency; CTLA4 = cytotoxic T-lymphocyte antigen 4; CVID = common variable immunodeficiency; DADA2 = deficiency of adenosine deaminase 2; DEB: Diepoxybutane; DNTs: double-negative T cells; eADA: erythrocyte adenosine deaminase; EBV = Epstein–Barr virus; FA: Fanconi anemia; FASL: FAS ligand; HLH = hemophagocytic lymphohistiocytosis; IgE = immunoglobulin E; IPEX: Immune Dysregulation, Polyendocrinopathy, Enteropathy, X-Linked Syndrome; LRBA = lipopolysaccharide-responsive beige-like anchor protein; pLPD = polyclonal lymphoproliferative disease; OS: Omenn syndrome; SAP: signaling lymphocyte activation molecule; STAT3/STAT1 = signal transducer and activator of transcription 3/1; Tregs: regulatory T cells; WAS: Wiskott–Aldrich syndrome; XIAP: X-linked inhibitor of apoptosis; XLA: X-linked agammabglobulinemia. The asterisk in panel (**E**) indicates that serum cytokines and other tests are performed based on clinical suspicion. If functional screening, disease staging, and other tests are negative, extended genetic testing is proceeded.

First-level exams should be tailored according to the presenting hematological phenotype and resource availability. Figure 4 provides an overview of the suggested investigations in patients presenting with hematological involvement and suspected IEI, according to the clinical phenotype. As a general rule, patients with AIC, pLPD, or malignancy should receive an assessment including complete blood count, proteinogram, serum immunoglobulin concentration, lymphocyte subpopulations, DNTs, and, when appropriate, ALPID-associated markers (IL-10, IL-18). Patients with BMF/MDS and high risk of IEI should receive a complete work-up to exclude IBMFs (including analysis of erythrocyte adenosine deaminase, chromosome fragility, telomere length, and others) [96] accompanied by an immunological assessment [97], while in patients presenting with eosinophilia, the analysis of serum IgE, platelet count with mean platelet volume, and lymphocyte absolute count can significantly orient the diagnostic process. Second-level investigations should be performed based on the diagnostic suspicion and include apoptotic assay (when suspecting ALPS), switched memory B cells and vaccine responses (when CVID is suspected), lymphocyte proliferation assay (for CID), CD57+ CD8+ T cells (typically elevated in APDS), CTLA-4 expression, STAT phosphorylation, and others [93]. When available, disease-specific biomarkers can orient towards a definitive diagnosis more rapidly than genetic testing and thus play a relevant role in stratifying patients for appropriate therapy, particularly in those who are eligible for targeted therapies.

Patients with HLH require specific considerations, as some rapid functional and genetic screening tests are available. These include flow cytometry for perforin expression or NK cell degranulation as well as targeted sequencing for known FHL genes [65]. Given that HLH and HLH mimics can occur in almost all IEI categories, a broader diagnostic approach through functional immune testing (i.e., serum cytokines, ADA2 activity, and others based on the clinical suspicion) and broad genetic analysis may be necessary in selected cases [73].

Beyond HLH, due to overlapping phenotypes, genetic testing—preferably via next-generation sequencing or whole exome sequencing panels—is often required to establish a definitive diagnosis in patients with IEI-associated hematological manifestations. Given that the genetic landscape of IEIs with immune dysregulation and hematological involvement is still partly unexplored, the interpretation of genetic testing remains significantly challenging. Therefore, it is strongly suggested to perform genetic panels in centers with high experience in the field of IEIs.

## 9. Conclusions

A wide range of IEIs can present with hematological involvement, and early recognition and genetic diagnosis are crucial for implementing life-saving, personalized therapies and improving patient outcomes. In order to achieve this, the identification of some clinical predictors of IEI in patients with hematological diseases is of pivotal importance to promptly refer patients to immunological assessment. Hopefully, a better characterization of IEIs with hematological phenotype, as well as the implementation of current guidelines with an emphasis on the identification of IEI-associated warning signs, will facilitate the timely identification and treatment of this heterogeneous group of conditions.

## Figures and Tables

**Table 1:** Most relevant IEIs associated with autoimmune cytopenia [10,12,13].

Immune dysregulation disorders	Autoimmune lymphoproliferative syndrome (ALPS-FAS, ALPS-FASL, ALPS-sFAS, ALPS-CASP10, ALPS-U) Other diseases of the ALPID spectrum: Disorders of Tregs: (CTLA4 and LRBA haploinsufficiency, STAT3 GOF, STAT1 GOF, BACH2 deficiency) Protein kinase C δ deficiency (PKCD) EBV-related disorders
Primary antibody deficiencies	CVID spectrum Activated phosphoinositide 3-kinase d syndrome (APDS 1, APDS2, APDS-L) Iper-IgM unPAD
Others	Syndromic IEIs (22q11.2DS, and others) Combined immunodeficiencies RAG-associated diseases (i.e., hypomorphic RAG1 and RAG2 mutations) Others (i.e., DADA2, Kabuki syndrome) Phenocopies of IEIs (i.e., RALD)

Abbreviations: IEIs = inborn errors of immunity; 22q11.2DS = 22q11.2 deletion syndrome; ALPS = autoimmune lymphoproliferative syndrome; ALPID = autoimmune lymphoproliferative immunodeficiencies; APDS = activated PI3K-delta syndrome; BACH2 = BTB domain and CNC homolog 2; CASP10 = caspase 10; CTLA4 = cytotoxic T-lymphocyte antigen 4; CVID = common variable immunodeficiency; DADA2 = deficiency of adenosine deaminase 2; EBV = Epstein–Barr virus; FAS = Fas cell surface death receptor; FASL = Fas ligand; LRBA = lipopolysaccharide-responsive beige-like anchor protein; PKCD = protein kinase C delta; RAG = recombination activating gene; RALD = RAS-associated autoimmune leukoproliferative disease; STAT3/STAT1 = signal transducer and activator of transcription 3/1; Treg = regulatory T cells; unPAD = unclassified primary antibody deficiency.

**Table 4:** Most relevant IEIs associated with HLH [52].

Primary HLH	Gene
**FHL without hypopigmentation**	
FHL-1	Unknown
FHL-2 (Perforin deficiency)	*PRF1*
FHL-3 (UNC13D deficiency)	*UNC13D*
FHL-4 (Syntaxin 11 deficiency)	*STX11*
FHL-5 (STXBP2 deficiency)	*STXBP2*
**FHL with hypopigmentation**	
Griscelli syndrome type 2	*RAB27A*
Chediak-Higashi syndrome	*LYST*
Hermansky-Pudlak syndrome type 2	*AP3B1*
Hermansky-Pudlak syndrome type 10	*AP3D1*
GIMAP6 deficiency	*GIMAP6*
**Other immune dysregulation disorders associated with HLH**	
XLP-1	*SH2D1A*
XLP-2	*BIRC4*
CD27 deficiency	*CD27*
CD137 deficiency	*CD137*
TIM3 deficiency	*HAVCR2*

Abbreviations: AP3B1 = adaptor-related protein complex 3 subunit beta 1; AP3D1 = adaptor-related protein complex 3 subunit delta 1; BIRC4 = baculoviral IAP repeat-containing protein 4; CD27: cluster of differentiation 27; CD137: cluster of differentiation 137; FHL = familial hemophagocytic lymphohistiocytosis; GIMAP6 = GTPase, IMAP family member 6; HAVCR2 = hepatitis A virus cellular receptor 2; HLH = hemophagocytic lymphohistiocytosis; LYST = lysosomal trafficking regulator; PRF1 = perforin 1; RAB27A = Ras-related protein Rab-27A; SH2D1A = SH2 domain containing 1A; STXBP2 = syntaxin binding protein 2; UNC13D = unc-13 homolog D; STX11 = syntaxin 11; TIM3 = T-cell immunoglobulin and mucin-domain containing-3; XLP-1 = X-linked lymphoproliferative syndrome type 1; XLP-2 = X-linked lymphoproliferative syndrome type 2.

## Data Availability

Not applicable.

## Abbreviations

ADA2	Adenosine Deaminase 2
AIC	Autoimmune Cytopenia
AIHA	Autoimmune Hemolytic Anemia
AIN	Autoimmune Neutropenia
ALPID	Autoimmune Lymphoproliferative Immunodeficiencies
AML	Acute Myeloid Leukemia
AP3B1	Adaptor-Related Protein Complex 3 Subunit Beta 1
AP3D1	Adaptor-Related Protein Complex 3 Subunit Delta 1
APDS	Activated PI3K-Delta Syndrome
AT	Ataxia–Telangiectasia
BIRC4	Baculoviral IAP Repeat Containing 4
BMF	Bone Marrow Failure
CASP10	Caspase 10
CD25	Cluster of Differentiation 25
CD27	Cluster of Differentiation 27
CD137	Cluster of Differentiation 137
CD57+ CD8+	Differentiated Cytotoxic CD8+ T Cells expressing CD57
CECR1	Cat Eye Syndrome Chromosome Region, Candidate 1
CID	Combined Immunodeficiency
CTLA4	Cytotoxic T-Lymphocyte Antigen 4
CVID	Common Variable Immunodeficiency
DADA2	Deficiency of Adenosine Deaminase 2
DNTs	Double-Negative T Cells
DNA	Deoxyribonucleic Acid
DOCK8	Dedicator of Cytokinesis 8
EBV	Epstein–Barr Virus
ES	Evans Syndrome
FAAP24	Fanconi Anemia-Associated Protein 24
FAS	Fas Cell Surface Death Receptor
FASL	Fas Ligand
FHL	Familial Hemophagocytic Lymphohistiocytosis
FOXP3	Forkhead Box P3
GATA2	GATA Binding Protein 2
GIMAP6	GTPase IMAP Family Member 6
GLILD	Granulomatous–Lymphocytic Interstitial Lung Disease
GOF	Gain of Function
H-ID	Hematologic Immune Dysregulation
HIES	Hyper-IgE Syndrome
HLH	Hemophagocytic Lymphohistiocytosis
HPV	Human Papillomavirus
HSCT	Hematopoietic Stem Cell Transplantation
IBMF	Inherited Bone Marrow Failure
IEIs	Inborn Errors of Immunity
IgA	Immunoglobulin A
IgE	Immunoglobulin E
IgG	Immunoglobulin G
IgM	Immunoglobulin M
IL-2	Interleukin 2
IL-10	Interleukin 10
IL-18	Interleukin 18
IPEX	Immune Dysregulation, Polyendocrinopathy, Enteropathy, X-Linked Syndrome
ITP	Immune Thrombocytopenia
ITK	IL-2-Inducible T-cell Kinase
IUIS	International Union of Immunological Societies
JAK	Janus Kinase
KRAS	Kirsten Rat Sarcoma Viral Oncogene Homolog
LOF	Loss of Function
LPD	Lymphoproliferative Disease
LRBA	Lipopolysaccharide-Responsive Beige-Like Anchor
LYST	Lysosomal Trafficking Regulator
MALT	Mucosa-Associated Lymphoid Tissue
MDS	Myelodysplastic Syndromes
MIRAGE Syndrome	Myelodysplasia, Infection, Restriction of Growth, Adrenal Hypoplasia, Genital Anomalies, Enteropathy
NFKB1	Nuclear Factor Kappa B Subunit 1
NHL	Non-Hodgkin Lymphoma
NK	Natural Killer
NRAS	Neuroblastoma RAS Viral Oncogene Homolog
OS	Omenn Syndrome
PET/CT	Positron Emission Tomography/Computed Tomography
PI3K	Phosphoinositide 3-Kinase
PI3Kδ	PI3K Delta Isoform
PKCD	Protein Kinase C Delta
pLPD	Polyclonal Lymphoproliferative Disease
PRF1	Perforin 1
RAB27A	RAS-Related Protein Rab-27A
RALD	RAS-Associated Autoimmune Leukoproliferative Disease
RASGRP1	RAS Guanyl Releasing Protein 1
RHOG	Ras Homolog Family Member G
SAM	Severe Dermatitis, Multiple Allergies, and Metabolic Wasting Syndrome
SAMD9/SAMD9L	Sterile Alpha Motif Domain Containing 9/Like
SCID	Severe Combined Immunodeficiency
SH2D1A	SH2 Domain Containing 1A
SLC7A7	Solute Carrier Family 7 Member 7
STAT1	Signal Transducer and Activator of Transcription 1
STAT3	Signal Transducer and Activator of Transcription 3
STAT5b	Signal Transducer and Activator of Transcription 5b
STK4	Serine/Threonine Kinase 4
STX1	Syntaxin 1
STXBP2	Syntaxin Binding Protein 2
TACI	Transmembrane Activator and CAML Interactor
T-LGLL	T-cell Large Granular Lymphocytic Leukemia
TIM3	T-cell Immunoglobulin and Mucin-domain Containing-3
Treg	Regulatory T Cells
UNC13D	Unc-13 Homolog D
unPAD	Unclassified Primary Antibody Deficiency
WAS	Wiskott–Aldrich Syndrome
XLP	X-Linked Lymphoproliferative Syndrome
XLP-1	X-Linked Lymphoproliferative Syndrome Type 1
XLP-2	X-Linked Lymphoproliferative Syndrome Type 2
XMEN	X-Linked Immunodeficiency with Magnesium Defect, Epstein–Barr Virus Infection, and Neoplasia
